# Wheat Grain Yield Increase in Response to Pre-Anthesis Foliar Application of 6-Benzylaminopurine Is Dependent on Floret Development

**DOI:** 10.1371/journal.pone.0156627

**Published:** 2016-06-03

**Authors:** Chunfeng Zheng, Yunji Zhu, Chenyang Wang, Tiancai Guo

**Affiliations:** 1 The College of Agronomy, Henan Agricultural University, Zhengzhou, 450002, China; 2 The Collaborative Innovation Centre of Henan Food Crops, Henan Agricultural University, Zhengzhou, 450002, China; 3 The National Engineering Research Centre for Wheat, Henan Agricultural University, Zhengzhou, 450002, China; Huazhong University of Science and Technology, CHINA

## Abstract

Wheat yield is largely determined during the period prior to flowering, when the final numbers of fertile florets and grains per spike are established. The aim of this study was to assess the dynamics of floret primordia development in winter wheat in response to pre-anthesis application of a synthetic cytokinin, 6-benzylaminopurine (6-BA). We conducted an experiment in which two foliar spray treatments were applied (water or 6-BA) to Chinese winter wheat at 25 days after jointing during two growing seasons (2012–2013 and 2013–2014). Both the final grain number per spike and grain yield at maturity exhibited remarkable increases in response to the 6-BA treatment. Application of 6-BA increased the number of fertile florets in basal spikelets and, to a greater extent, in central spikelets. The mechanism by which 6-BA application affected the final number of fertile florets primarily involved suppression of the floret abortion rates. Application of 6-BA considerably reduced the abortion rates of basal, central and apical spikelet florets (by as much as 77% compared with the control), as well as the degeneration rates of basal and central spikelet florets, albeit to a lesser degree. The effect of 6-BA application on the likelihood of proximal florets being set was limited to the distal florets in the whole spike, whereas obvious increases in the likelihood of grain set under 6-BA treatment were observed in distal florets, primarily in central spikelet positions. The results of this study provide important evidence that 6-BA application to florets (final fertile floret production) results in an increased grain yield.

## Introduction

To further increase wheat yield, it may be useful to elucidate the mechanisms controlling yield determination [1,2]. In analyses of the physiological determinants of cereal productivity, yield is commonly divided into its two major components: the number of grains set per ground area and the average grain weight. Because grain growth is often restricted by sink rather than source tissues [3–5], the yields of wheat and most other crops are primarily influenced by the grain number per unit area [6–10].

The potential grain number is determined during the pre-anthesis stage [11] as a result of the intricate processes of floret generation and degeneration. First, floret structures, which later become fertile and capable of bearing grains, are generated. A large proportion of these structures then degenerate, and a small proportion of them subsequently abort (a period of fertile floret degeneration) [12,13]. Most of these processes occur when the spikes are attached along the culm during the pre-anthesis period. Grain number is related to the number of fertile florets [14] during the stem elongation phase. The number of fertile florets is most frequently determined by the survival of florets rather than by the number of florets produced [15].

Many reports in the literature have assumed that floret development depends on resource availability in wheat. González et al. [16] and Ferrante et al. [17] have shown that the numbers of both grains and fertile florets are related to the spike dry weight at anthesis. González et al. [16] have found that floret death and survival are linked to pre-anthesis spike growth. Floret death occurs during rapid stem and spike elongation, at a time when nutrient requirements are high. Therefore, it is frequently argued that competition for assimilates eventually leads to a shortage of nutrients, resulting in floret primordia abortion. Ferrante et al. [12] have demonstrated that nitrogen and water availabilities affect the rate of floret development and therefore the rate of floret primordia mortality in one cultivar of durum wheat. Many studies have also reported on the effects of N application timing and management on yield [18,19] and on the developmental dynamics of florets in response to N availability [12,15]. For example, Zhang et al. [20] have reported that both plant growth regulators and N application timing markedly affect floret initiation and development. Several studies have also described different factors affecting yield and crop parameters after the spraying of plant growth regulators of wheat [21–24]. Moreover, Serrago et al. [25] have identified a relationship between the fate of individual florets, which determines floret survival, and spike growth dynamics, as affected by photoperiod manipulation during stem elongation. However, to our knowledge, the relationship between floret primordia development (specifically, the final number of fertile florets produced) and grain yield in response to the pre-anthesis foliar application of a synthetic cytokinin, 6-benzylaminopurine (6-BA), in wheat remains unclear.

Therefore, the aim of this study was to measure the effects of the pre-anthesis foliar application of 6-BA on yield and grain number in well-adapted winter wheat and to determine whether the responses are related to the developmental dynamics of florets, specifically to the final production of fertile florets.

## Materials and Methods

### Plant materials

The common wheat cultivar Yumai 49–198 was used in this study, as its performance has been previously demonstrated under field conditions, e.g., in a study conducted by Zheng et al. [13], in which it was grown at a farm at the Science and Technology Demonstration Park of Henan Agricultural University (113° 59′ E, 34° 86′ N, Zhengzhou, Henan Province, China) during the 2012–2013 and 2013–2014 growing seasons. The soil was light loam, and the basic chemical properties of the cultivated layer at 0 to 20 cm depth before sowing were as follows: 16.8 g kg^-1^ organic matter, 0.9 g kg^-1^ total N, 25.6 mg kg^-1^ available phosphorus, 124.5 mg kg^-1^ available potassium, 0.41 mg kg^-1^ available B, and a pH of 8.62. Before sowing, all experimental plots were fertilized with 75 kg phosphate (P_2_O_5_), 60 kg potash (K_2_O), and 73 kg nitrogen (using urea, 46%) as a basal application, and the same quantities of fertilizers were topdressed at the jointing stage. The seeds were sown on 8 October during the two growth periods. Each experimental unit consisted of a 20 m^2^ plot with row spacing of 20 cm and 225 plants/m^2^. The experiment was arranged in a randomized block design. Plots were sprayed with either water or 6-BA (10 mg/L) at 25 days after the jointing stage (prior to the abortion of florets). The amount of 6-BA sprayed was controlled following the protocol described by Shi et al. [26], which entails the drip-free spraying of water on the surfaces of leaves. Each treatment was replicated three times.

### Sampling and measurements

**Fig 1:**
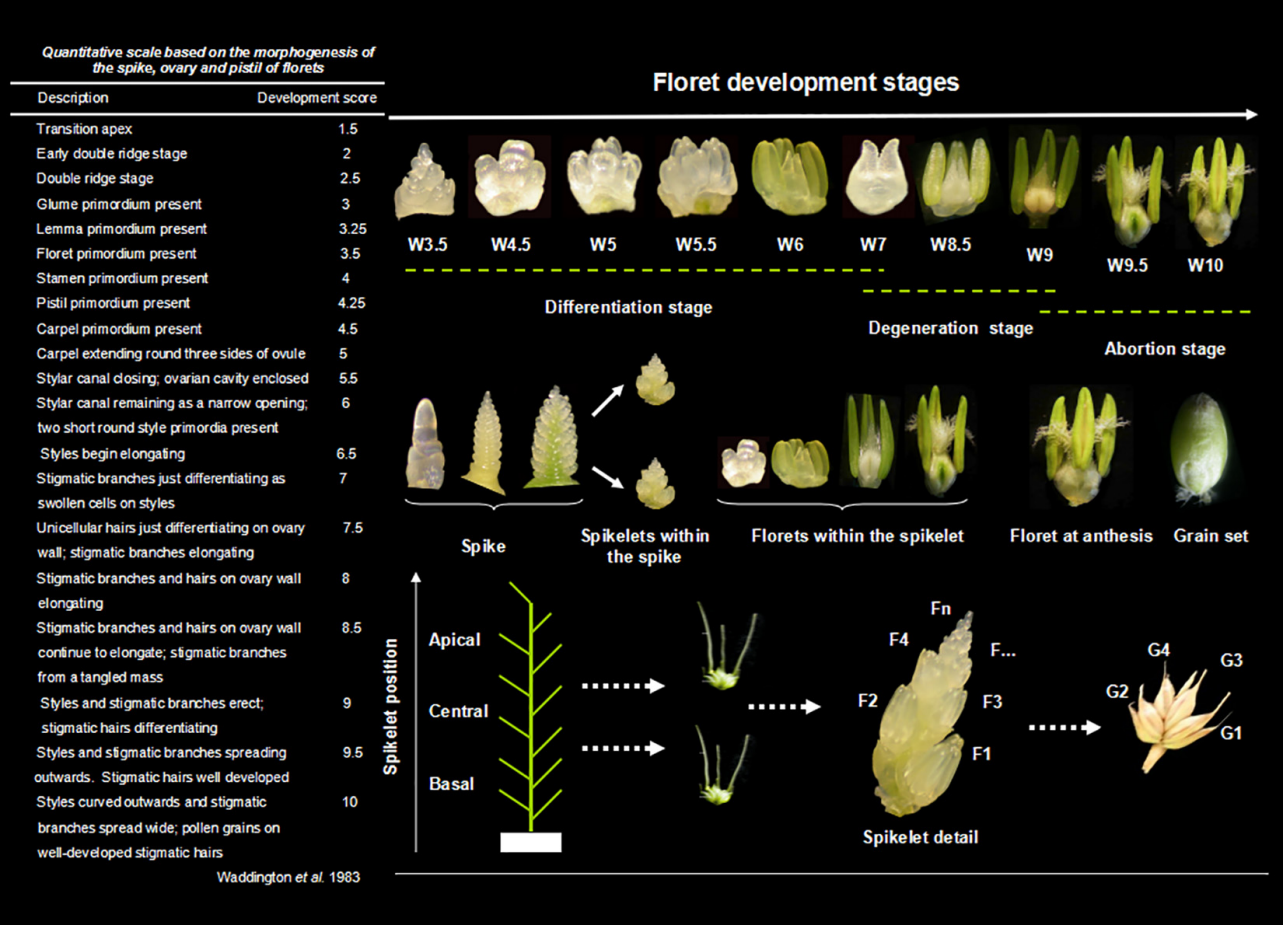
Development of the floret primordia. Floret developmental stages were determined using the Waddington scale [28] (left panel), with pictures captured using a stereomicroscope (XTZ-E BM (180x)). The panel on the upper right side illustrates floret developmental dynamics in three stages: differentiation (from the first appearance of floret primordium [W3.5] to the end of floret initiation [W7]), degeneration (i.e., the tetrad stage of floret development, from W7 to W9), and abortion (from pollen maturity [W9] to grain setting). The central panel first depicts the development of spikelets within the spike. Second, the development of floret primordia within spikelets is presented. Third, a floret successfully reaching the final fertile stage at flowering (W10) and grain setting is presented. The bottom panel illustrates the spikelet positions within the spike (left), the florets within spikelets from those closest to the rachis (F1) to those located at increasingly distal positions (middle), and the set grains numbered according to their positions relative to rachis, from G1 (the most proximal) to G4 (the most distal) (right). The drawings and pictures are not to scale: as a reference, the floret widths are approximately 0.15 mm in W3.5, 0.20 mm in W5, 0.50 mm in W7, 0.90 mm in W8.5, and 2.20 mm in W10.

Five plants from each experimental unit (15 plants per treatment) were randomly harvested weekly. Samples were obtained every three days from the onset of spraying with 6-BA or water until five days after flowering. The main shoot was dissected under a stereomicroscope (XTZ-E BM [180x], BM Optical Instruments Manufactory, Shanghai, China), and the following measurements were recorded: the differentiation stage of young spikes, the number of differentiated spikelets, the total number of floret primordia, and the morphological characteristics of florets at the different developmental stages. In addition, the developmental stages of the florets were determined. The spikelets considered included those in the apical (first/fourth from the tip), central (middle), and basal (first/fourth from the base) positions of the spikes. We numbered the florets according to their positions relative to the rachis [27], ranging from Floret 1 (F1, closest to the rachis) to Fn (the most distal primordium), and the development of each floret was scored (Fig 1) using the Waddington scale [28], which assigns scores primarily according to pistil development from floret primordium present (stage W3.5) to stage W10. Fertile florets were defined as those at W8.5 or immediately before that stage (when the stigmatic branches formed a tangled mass) [28,29]. Florets at W10 were identified as ‘final fertile florets’ (Fig 1).

At maturity, 20 spikes were randomly harvested from the main shoots of the plants in each experimental unit. In each spike, the spikelets were separated, and the grains in each spikelet were numbered according to their positions relative to the rachis (from F1 at the most proximal site to F4 at the most distal site).

The growing degree days after sowing (GDD) was calculated as the summation of daily average temperature [(T_max_+T_min_)/2] (a base temperature of 0°C was assumed). To determine the dynamics of floret development during pre-anthesis, the numbers of living florets in successive samples across floret development stages were plotted against the GDD, and the data were fitted to a tri-linear model (a rising linear, sharply linear, and slowly dropping linear). The different developmental rates (differentiation, degeneration, and abortion rates) of the floret primordia were calculated using the three linear equations of the dynamic model of floret development, *i*.*e*., the slope of the equation was the floret developmental rate.

### Climate variations during growth periods

**Fig 2:**
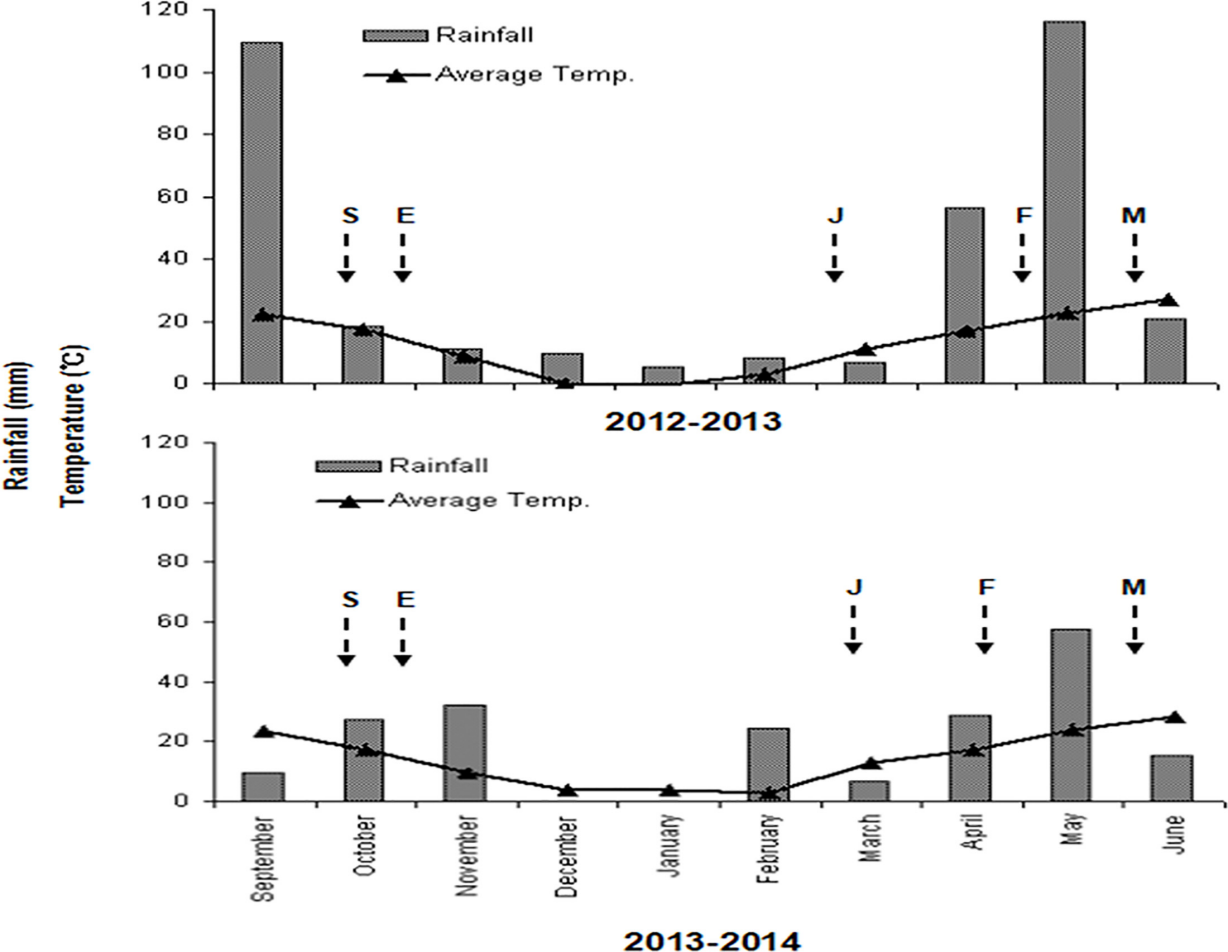
Accumulated rainfall (bars) and the mean temperatures (points and line) over the 2012–2013 and 2013–2014 wheat-growing periods at the experimental field (Zhengzhou, China). The arrows denote the dates: sowing stage (S), seeding emergence stage (E), jointing stage (J), anthesis stage (F), and maturity stage (M) for wheat.

The climatic conditions varied between the 2012–2013 and 2013–2014 growing periods. The 2013–2014 growing period experienced higher temperatures and less rainfall (Fig 2). In 2013–2014, a serious drought occurred in this region during pre-flowering, resulting in 27.5 mm less rainfall from the jointing to the flowering stages compared with that in 2012–2013. From December 2013 to the end of January 2014, the rainfall amounted to merely 0.1 mm, which was 15 mm less than the average rainfall in 2012–2013. From November 2013 to March 2014, the monthly average temperature was 1.12°C higher than that during the same period in 2012–2013. The total accumulated temperature during the entire 2013–2014 growing period was 380.7°C higher than that during the 2012–2013 growing period.

### Statistical analysis

All data were assessed by analysis of variance (ANOVA), performed using SPSS (version 17.0), to examine differences between the two spraying treatments. Least significant differences (LSDs) were calculated at a probability level of P = 0.05. Figures were created using Microsoft Excel 2003.

## Results

### Effect of 6-BA application on wheat grain yield

As expected, the foliar application of 6-BA resulted in increased grain yields at maturity during both the 2012–2013 and 2013–2014 growing periods (Table 1). Compared with the plants treated with the water control, the grain yields of the 6-BA-treated plants increased by 5.63% from 2012–2013 and by 8.9% from 2013–2014. The grain numbers per spike were significantly increased in response to the 6-BA treatment compared with the water treatment during both growing periods; however, no differences in thousand-grain weight were noted (Table 1). The grain number per spike and thousand-grain weight increased by 3.252 grains and 0.83 g, respectively, in response to the 6-BA treatment in 2012–2013, and these values increased by 3.81 grains and 0.99 g, respectively, in 2013–2014.

**Table 1:** Grain yields and main yield components of winter wheat between the two treatments for the two growing periods.

Growing period	Treatment	Spikes (10^4^ ha^-1^)	Grains per spike	1000-grain weight (g)	Grain yield (kg ha^-1^)
	Water	625.11±13.99a	31.92±1.53b	40.22±1.95a	7991.27±53.06b
2012–2013	6-BA	625.4±14.29a	35.17±0.52a	41.05±1.004a	8440.87±93.71a
	MS	0.126NS	21.206*	1.025NS	349836.907**
	Water	690.88±15.39a	26.45±1.48b	52.34±1.09a	8077.79±31.41b
2013–2014	6-BA	690.2±13.01a	30.26±1.46a	53.33±0.14a	8797.25±67.53a
	MS	0.960NS	30.061*	1.17NS	639208.704***

*Note*: The values are the mean ± standard error, n = 3. Different letters in the same column indicate significant differences between treatments at a P < 0.05. The mean square (MS) for the effect of foliar spraying of 6-BA is also presented.

*, **, ***, and NS represent the levels of significance of the MS values (0.05, 0.01, 0.001, and non-significant, respectively).

### Effect of 6-BA application on living floret dynamics

**Fig 3:**
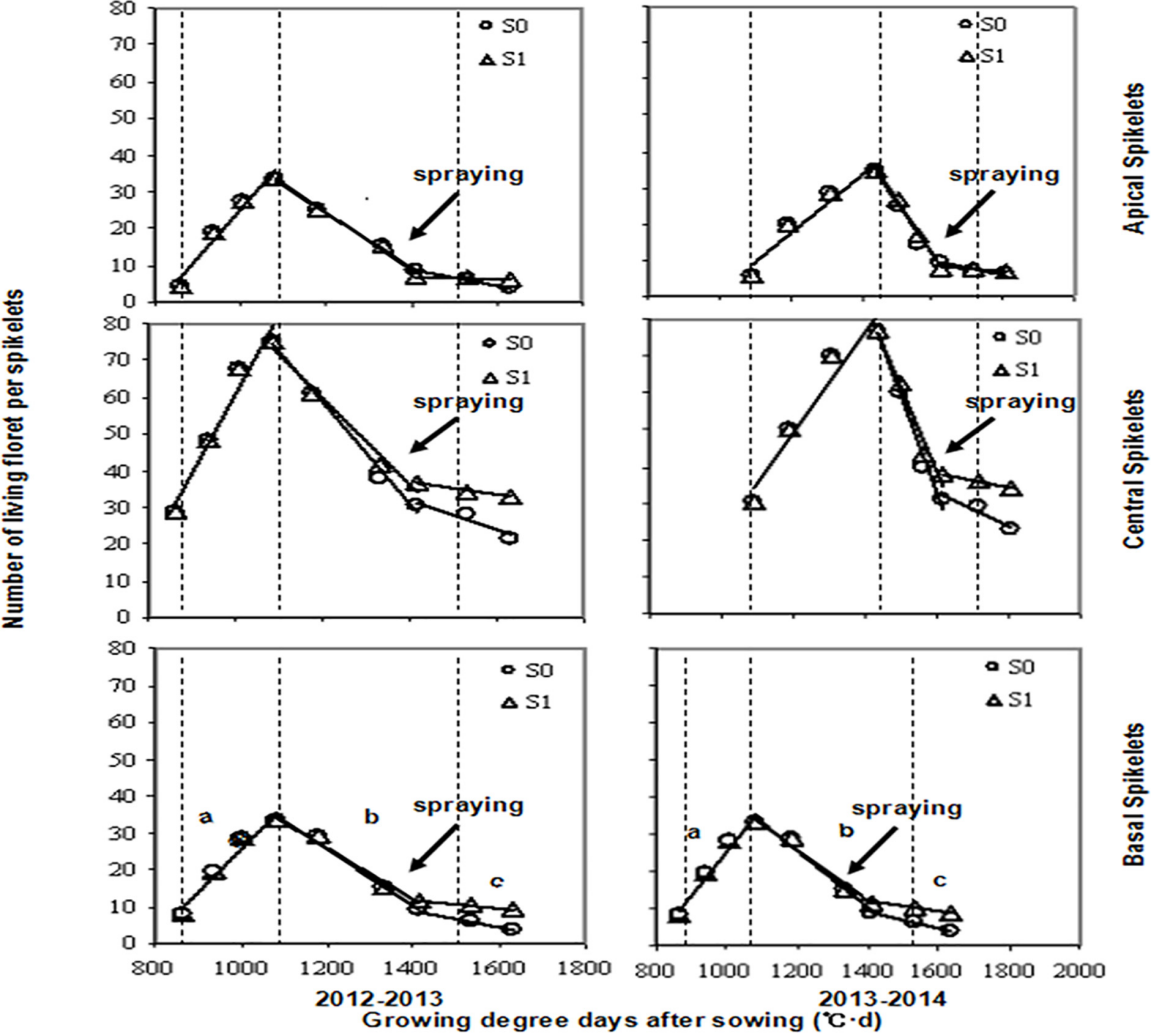
Dynamics of floret primordia from jointing to five days after anthesis in winter wheat in response to two foliar spray treatments (S0: water [circles]; S1: 6-BA [triangles]) in three spikelet categories (basal, central and apical). The lowercase letters indicate the three stages of floret developmental dynamics: differentiation (a), degeneration (b) and abortion (c). In each panel, the left dotted lines indicate jointing, the middle dotted lines indicate 21 days after jointing, and the right dotted lines indicate flowering. The arrows indicate the point at which spraying was conducted.

The dynamics of living florets subjected to the spraying treatment showed similar patterns during both growing periods (Fig 3). The model of floret development was characterized by three stages: first, the floret primordia number increased until a maximum was reached at the peak (approximately 35 floret primordia on basal and apical spikelets, and approximately 80 floret primordia on central spikelets); second, a period of floret degeneration occurred with the highest rate of primordia loss; and, third, a final stage of floret abortion occurred, during which the rate of degeneration diminished but the differences between treatments were the greatest (Fig 3).

During the final periods of floret development in both growing periods, the foliar application of 6-BA increased the numbers of fertile florets in the basal and central spikelets, whereas a minimal effect on the apical spikelet was noted (Fig 3). Moreover, the number of fertile florets exhibited a stronger increase in response to 6-BA spraying in the central compared with the basal spikelet, whereas the number of fertile florets only increased slightly (Fig 3).

Significant differences in floret development rates were noted between the two treatments (Table 2). During both growing periods, the floret abortion rates decreased in the basal, central and apical spikelets after 6-BA spraying compared with the water control treatment (Table 2) by 72.22%, 76.81% and 66.67% in 2012–2013 and by 59.11%, 73.64% and 45.83% in 2013–2014, respectively. Further, the floret degeneration rates in basal and central spikelets were reduced after 6-BA spraying (Table 2) by 10.87% and 22.86% in 2012–2013 and by 9.6% and 21.23% in 2013–2014, respectively. However, foliar application of 6-BA did not affect the degeneration rate of florets from apical spikelets (Table 2).

**Table 2:** Developmental rates of floret primordia for the two treatments at different spikelet positions over the two growth periods.

Growing period	Spikelet position	Treatment	Differentiation rate	Degeneration rate	Abortion rate
	Basal	Water	0.1158±0.002a	0.092±0.007a	0.045±0.011a
		6-BA	0.1158±0.002a	0.082±0.003b	0.0125±0.007b
2012–2013	Central	Water	0.2177±0.003a	0.14±0.004a	0.069±0.002a
		6-BA	0.2177±0.003a	0.108±0.005b	0.016±0.005b
	Apical	Water	0.1325±0.0029a	0.073±0.006a	0.018±0.003a
		6-BA	0.1325±0.0029a	0.082±0.001a	0.006±0.01b
	Basal	Water	0.1780±0.0022a	0.1405±0.007a	0.0269±0.005a
		6-BA	0.1780±0.0022a	0.127±0.005b	0.011±0.003b
2013–2014	Central	Water	0.2879±0.0029a	0.1592±0.004a	0.0459±0.002a
		6-BA	0.2879±0.0029a	0.1254±0.003b	0.0121±0.001b
	Apical	Water	0.1580±0.0016a	0.1406±0.006a	0.024±0.003a
		6-BA	0.1580±0.0016a	0.1417±0.007a	0.013±0.008b

*Note*: The values are the mean ± standard error (±SE), n = 3. Different letters in the same column indicate significant differences between treatments at a P < 0.05.

### Effect of 6-BA treatment on grain setting

**Fig 4:**
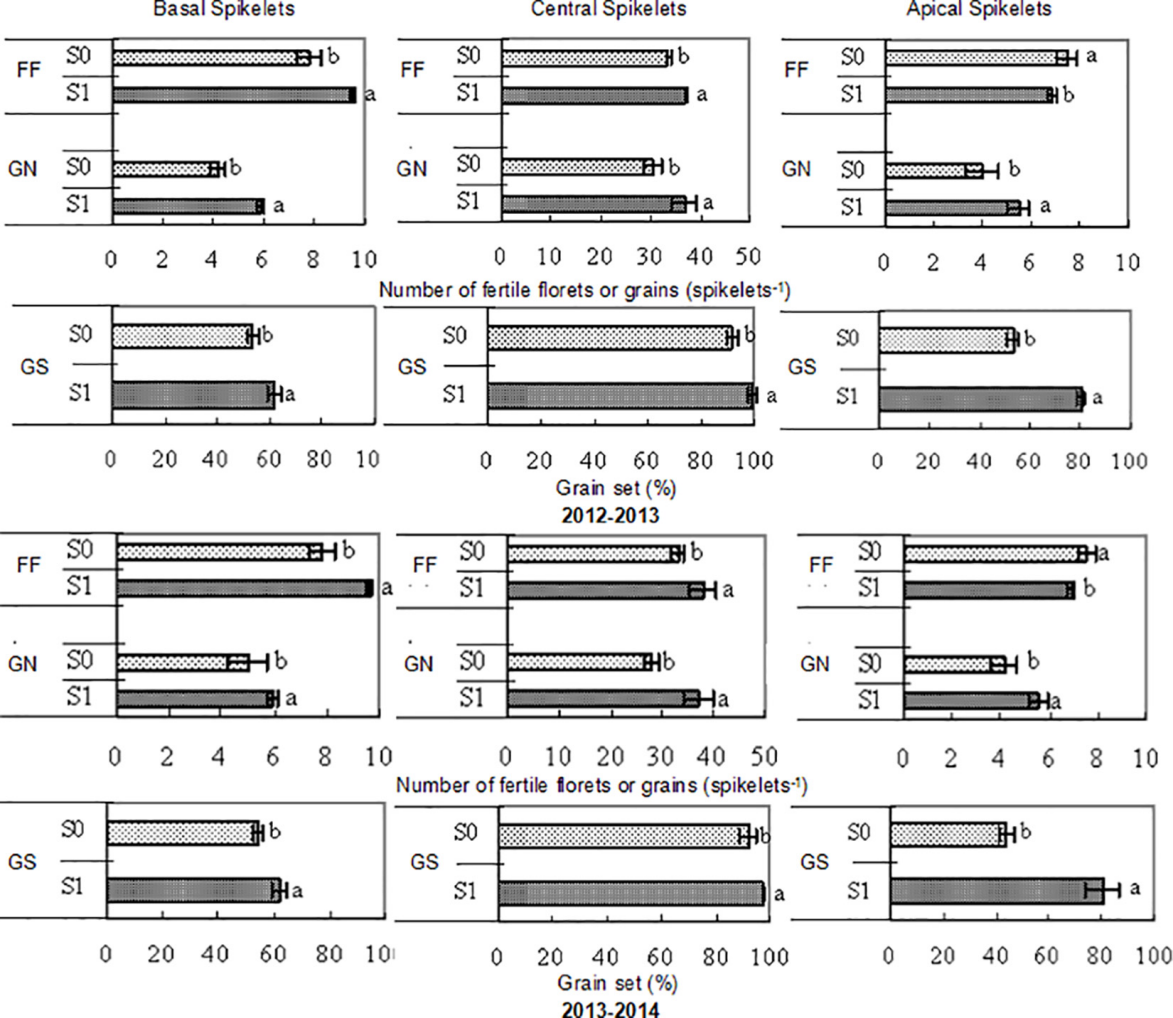
The number of fertile florets at anthesis (FF), grain number at maturity (GN), and percentage of grain set (GS) in winter wheat in response to two foliar spray treatments (S0: water [pale grey bars]; S1: 6-BA [dark grey bars]) in three spikelet categories (basal, central and apical). The data are presented as the treatment mean ± standard error, n = 3. The different letters indicate significant differences at a P < 0.05.

The application of 6-BA had remarkable effects on the final grain number at maturity in all spikelet positions. During both growing periods, the final grain number and grain setting rate of fertile florets increased in response to the 6-BA treatment compared with the water control treatment in the basal, central and apical spikelet positions (Fig 4). In other words, the responsiveness of grain number to 6-BA application was mainly observed as an increase in the grain setting rate of fertile florets. In the central and basal spikelet positions, the number of fertile florets increased in response to the 6-BA treatment compared with the water control treatment during both growing periods, whereas this number increased in response to the water control treatment compared with the 6-BA treatment in the apical spikelet position (Fig 4).

**Fig 5:**
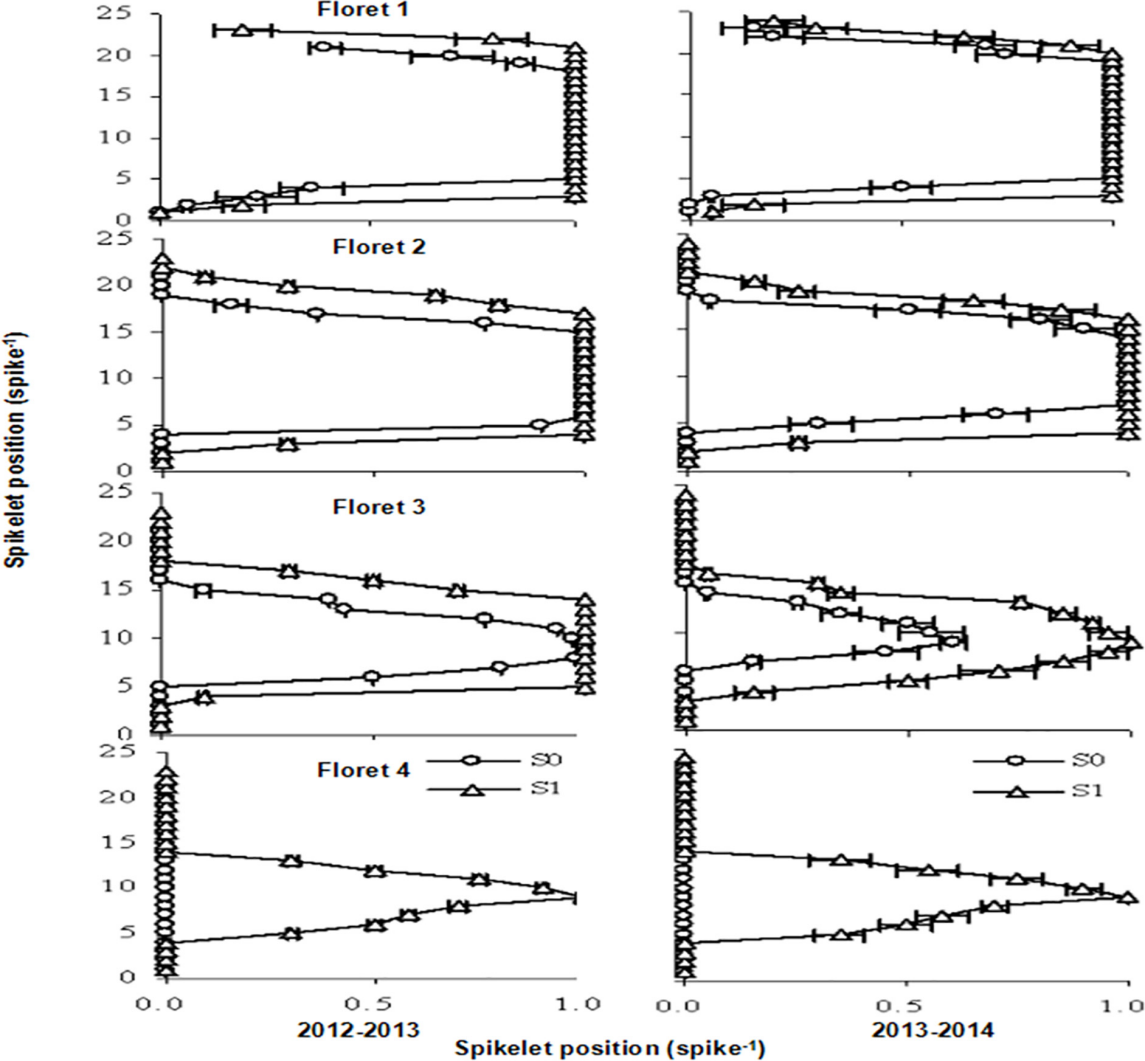
Effect of foliar spray treatment (S0: water [circles]; S1: 6-BA [triangles]) on likelihood of grain set in different floret positions (from F1 to F4) for different spikelet positions throughout the spike.

The most proximal florets usually set grains in a large proportion of spikelets, and the probability of a grain being set was generally higher in florets that were close to the rachis, i.e., ranging from the highest probability in F1 to the lowest in F4. The effects of 6-BA application on the likelihood of F1 and F2 being set were restrained to that of the most distal floret being set (i.e., F3 and F4) in the whole spike during both growing periods (Fig 5). In contrast, 6-BA application increased the likelihood of F3 and F4 (florets at the most distal positions) being set across all spikelet positions (Fig 5).

**Fig 6:**
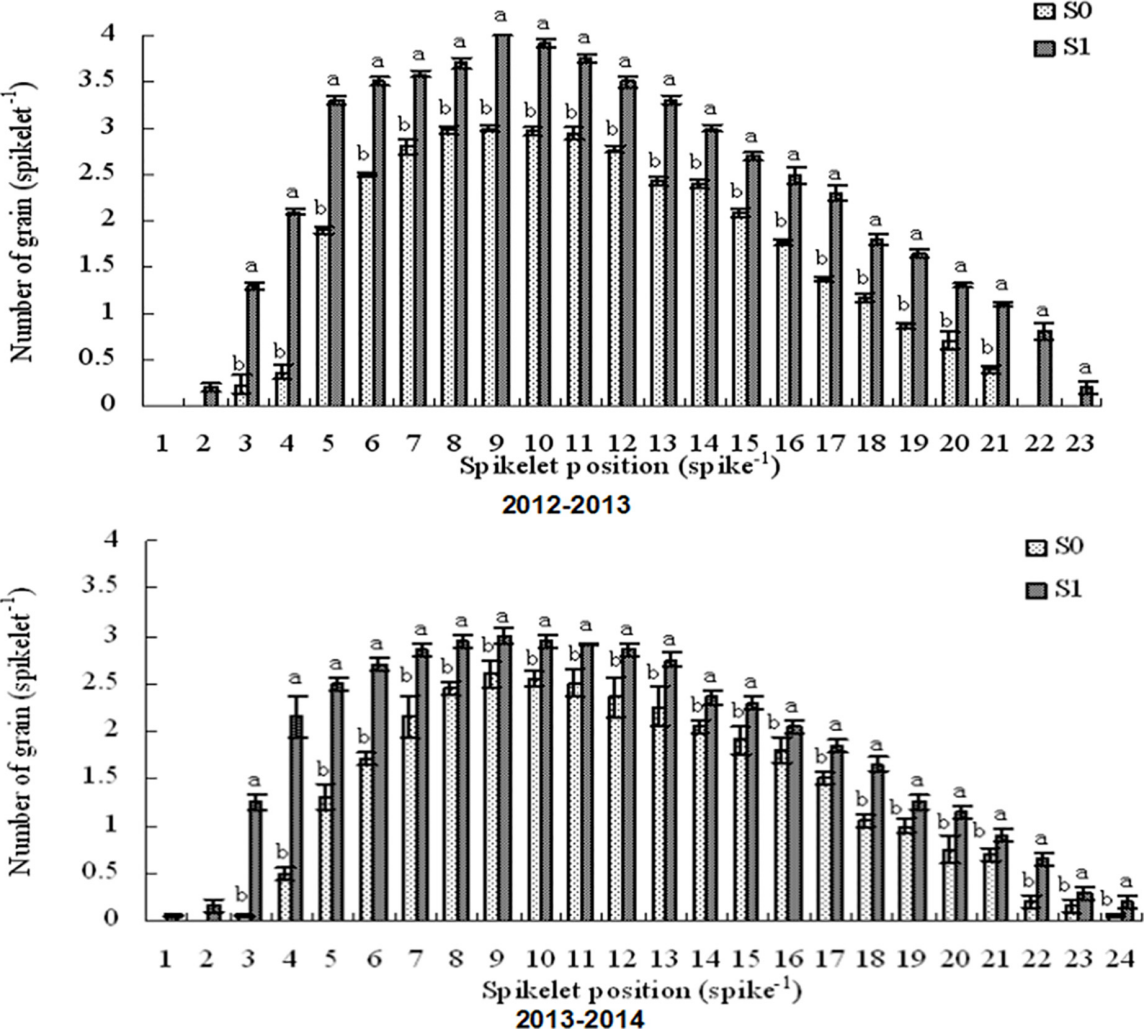
Number of grains at each spikelet position after the two foliar spray treatments (S0: water [pale grey bars]; S1: 6-BA [dark grey bars]). The data are presented as the treatment mean ± standard error, n = 3. The different letters indicate significant differences (P < 0.05) at each spikelet position.

The 6-BA treatment markedly affected the final grain number of spikelets during the two growth periods (2012–2013 and 2013–2014) (Fig 6). Following the foliar application of 6-BA, grain setting was initiated from the first or second spikelet position. and a total of 22 to 24 spikelets exhibited grain setting. In contrast, following the water control treatment, grain setting was initiated from the third spikelet position, and it was observed in only 21 to 22 spikelets (Fig 6). During both growing periods, the relative numbers of grain setting spikelets along the spikes were increased in response to the 6-BA treatment compared with the water control treatment (Fig 6).

## Discussion

### Dynamics of living florets

Several studies have reported that floret development in wheat involves a three-stage process, including generation, degeneration, and abortion (e.g., Li et al. [29]). Li et al. [30] have investigated the dynamics of floret differentiation and degeneration in winter and spring wheat. They have found that the relationship between the number of florets on the main stem and the GDD after sowing can be fitted using a quadratic equation, which consists of an accelerating curve, a promptly decelerating line, and a more gradually decelerating line thereafter. In our study, to elucidate the developmental dynamics of florets during pre-anthesis, the relationship between the number of living florets and the GDD was fitted using a tri-linear model, consisting of an increasing linear model (differentiation stage), a sharply decreasing linear model (degeneration stage), and a slowly decreasing linear model (abortion stage) (Fig 3). This model is more convenient for the calculation of the floret development rate. Due to the severe drought that occurred during the floret development stage of wheat growth in 2013–2014, the accumulated temperature at the onset of floret differentiation, degeneration and abortion in apical and central spikelets was almost 200°C higher than that in 2012–2013; however, this difference did not affect dynamics at the basal spikelet (Figs 2 and 3). Referring to Waddington’s scale [28], Ferrante et al. [15] have suggested that florets at W10 are fertile. However, in our study, we defined florets as fertile when they were at W8.5, consistent with Li et al. [29], after which a small proportion abort. In addition, we defined florets at W10 as 'final fertile florets'.

### Floret developmental stage and fertile floret survival

Floret primordia develop during a constrained time window, primarily coinciding with stem elongation [16]. Many scholars [16,31] have noted that rapid growth of the spike and stem during pre-anthesis leads to intense competition for limited nutrient resources. This competition results in a limited supply of nutrients for spike growth, resulting in floret degeneration. Some scholars have also indicated that at 15 d before anthesis, the spike:stem ratio reflects a stage with the highest level of competition and that the floret markedly degenerates during this stage [32,14]. Therefore, the final stage of the stem elongation phase is considered a critical period for fertile floret, grain number and yield determination [9].

However, Wang et al. [33,34] have found that plant hormones play important roles in floret development and that the effects of exogenous hormones on regulation of floret development are dependent on the developmental stage at which they are applied. Peltonen-Sainio [35] have found that exogenous zeatin (ZT) treatment significantly increases the grain number at the anther separation stage.

Here, we demonstrated that the foliar application of 6-BA during the late phase of floret development led to an increase in the grain number per spike (Figs 4 and 6). In response to the 6-BA treatment, the floret abortion rates in the basal, central and apical spikelets were reduced by 59.11–72.22%, 73.64–76.81% and 45.83–66.67% during the periods from 2012–2013 and from 2013–2014, respectively, compared with the water control treatment (Table 2). Thus, this study has confirmed that fertile floret survival is mediated by exogenous 6-BA during pre-anthesis (prior to the degeneration peak of florets).

### Fertile floret survival and grain yield

As yield is linearly related to grain number, the mechanisms underlying grain number determination may be relevant to the development of measures for increasing yield [1,6]. It has been subsequently established that the survival of fertile florets at anthesis under a wide range of conditions is closely related to the grain number at maturity, possibly due to genotypic variation [36] or to environmental influences [25,32]. Therefore, quantitative analysis of the dynamics of floret development may be important in investigations of the mechanisms for determining grain number and yield [37,38].

In the current study, foliar application of 6-BA at 25 days after the jointing stage (prior to the degeneration peak of common wheat florets) resulted in marked increases in the final grain number and grain yield at maturity but did not affect the thousand-grain weight. These results suggest that the striking effect of 6-BA application on wheat yield is more likely due to its effect on the grain number per spike than to that on the average grain weight. The response to 6-BA application was mainly attributed to an increase in the proportion of fertile florets exhibiting successful grain setting (Fig 4). The grain setting rates of fertile florets treated with 6-BA in the basal, central and apical spikelets increased by 8.11–10.55%, 12.64–15.06% and 5.43–8.35% during the 2012–2013 and 2013–2014 periods, respectively, compared with those treated with the water control (Fig 4). These results are consistent with a previous exploratory study reporting that the increased number of fertile florets during the late phase of floret development is driven by survival of the initiated florets [12].

### Possible causes of yield increases

Floret development is the result of a regulated balance between organ initiation and coordination on the one hand and meristem size on the other. Floral meristem size is controlled by auxin, gibberellin, and cytokinin, all of which play primary roles in organogenesis and organ initiation [39]. Cytokinins are hormones that regulate many physiological and developmental processes in plants [26,40], including generative development and grain yield [41–44]. In this study, we found that foliar application of the synthetic cytokinin 6-BA resulted in clear increases in the likelihood of grains set in distal florets (i.e., F3 and F4) in central spikelets. A previous study has demonstrated that cytokinins may affect yield by up-regulating genes associated with the cell cycle, resulting in increased seed size due to changes in sugar signalling and specifically to enhanced phloem unloading and sugar import into the endosperm through enzymatic activities of cell wall invertases [44]. Therefore, we hypothesize that that application of the exogenous hormone 6-BA during the late stage of floret development may change the levels of endogenous hormones in leaves and spikelets during the floret development process. Another possibility is that 6-BA application changes the distribution and supply of photosynthate to different positions of the wheat grain or spikelet, which could be favourable for development and grain setting in distal florets and spikelets. The internal physiological mechanism of the development of florets into grains, as mediated by exogenous 6-BA, requires further study.

## Conclusions

In this study, the foliar spraying of exogenous 6-BA (10 mg/L) prior to the peak in degeneration of wheat florets resulted in clear increases in grain number and yield in Chinese winter wheat. We therefore conclude that 6-BA application is an efficient strategy for improving cereal yields. We also demonstrated that the foliar spraying of 6-BA improved the final grain number and the fertile florets’ grain setting rate per spike by suppressing the abortion rate of fertile florets at any spikelet position of the spike. These findings confirm that the survival of fertile florets is a crucial determinant of grain number in wheat and that this process seemingly depends on resource availability (exogenous 6-BA) during pre-anthesis.

## Supporting Information

S1 Dataset Relevant data underlying the findings described in this manuscript. (XLS)

## References

[1] Fischer RA. Wheat physiology: a review of recent developments. Crop Pasture Sci. 2011;62: 95–114. 10.1071/CP10344

[2] Reynolds M, Foulkes J, Furbank R, Griffiths S, King J, Murchie E, et al. Achieving yield gains in wheat. Plant Cell Environ. 2012;35: 1799–1823. 10.1111/j.1365-3040.2012.02588.x 22860982

[3] Acreche MM, Slafer GA. Grain weight response to increases in number of grains in wheat in a Mediterranean area. Field Crops Res. 2006;98: 52–59. 10.1016/j.fcr.2005.12.005

[4] Bingham IJ, Blake J, Foulkes MJ, Spink J. Is barley yield in the UK sink limited? II. Factors affecting potential grain size. Field Crops Res. 2007;101: 211–220.

[5] Reynolds MP, Pellegrineschi A, Skovmand B. Sink-limitation to yield and biomass: a summary of some investigations in spring wheat. Ann Appl Biol. 2005;146: 39–49. 10.1111/j.1744-7348.2005.03100.x

[6] Pedro A, Savin R, Parry MAJ, Slafer GA. Selection for high grain number per unit stem length through four generations from mutants in a durum wheat population to increase yields of individual plants and crops. Field Crops Res. 2012;129: 59–70. 10.1016/j.fcr.2012.01.016

[7] Slafer GA. Genetic basis of yield as viewed from a crop physiologist’s perspective. Ann Appl Biol. 2003;142: 117–128. 10.1111/j.1744-7348.2003.tb00237.x

[8] Slafer GA, Gonzalez FG, Kantolic AG, Whitechurch EM, Abeledo LG, Miralles DJ, et al. Grain number determination in major grain crops In: Basra AS, editor. Handbook of seed science and technology. New York: Haworth Press; 2006 pp. 95–123.

[9] Fischer RA. The importance of grain or kernel number in wheat: a reply to Sinclair and Jamieson. Field Crops Res. 2008;105: 15–21. 10.1016/j.fcr.2007.04.002

[10] Reynolds M, Foulkes MJ, Slafer GA, Berry P, Parry MA, Snape JW, et al. Raising yield potential in wheat. J Exp Bot. 2009;60: 1899–1918. 10.1093/jxb/erp016 19363203

[11] Slafer G, Rawson H. Sensitivity of wheat phasic development to major environmental factors: a re-examination of some assumptions made by physiologists and modellers. Aust J Plant Physiol. 1994;21: 393–426. 10.1071/PP9940393

[12] Ferrante A, Savin R, Slafer GA. Floret development of durum wheat in response to nitrogen availability. J Exp Bot. 2010;61: 4351–4359. 10.1093/jxb/erq236 20696655 PMC2955747

[13] Zheng C, Zhu Y, Zhu HJ, Kang G, Guo T, Wang C. Floret development and grain setting characteristics in winter wheat in response to pre-anthesis foliar applications of 6-benzylaminopurine and boron. Field Crops Res. 2014;169: 70–76. 10.1016/j.fcr.2014.09.005

[14] Miralles DJ, Richards RA, Slafer GA. Duration of the stem elongation period influences the number of fertile florets in wheat and barley. Aust J Plant Physiol. 2000;27: 931–940.

[15] Ferrante A, Savin R, Slafer GA. Floret development and grain setting differences between modern durum wheats under contrasting nitrogen availability. J Exp Bot. 2013;64: 169–184. 10.1093/jxb/ers320 23162124 PMC3528029

[16] González FG, Miralles DJ, Slafer GA. Wheat floret survival as related to pre-anthesis spike growth. J Exp Bot. 2011;62: 4889–4901. 10.1093/jxb/err182 21705386

[17] Ferrante A, Savin R, Slafer GA. Differences in yield physiology between modern, well adapted durum wheat, cultivars grown under contrasting conditions. Field Crops Res. 2012;136: 52–64.

[18] González FG, Slafer GA, Miralles DJ. Grain and floret number in response to photoperiod during stem elongation in fully and slightly vernalized wheats. Field Crops Res. 2003;81: 17–27. 10.1016/S0378-4290(02)00195-8

[19] Fageria NK, Baligar VC. Enhancing nitrogen use efficiency in crop plants. Adv Agron. 2005;88: 97–185. 10.1016/S0065-2113(05)88004-6

[20] Zhang G, Chen J, Bull DA. The effects of timing of N application and plant growth regulators on morphogenesis and yield formation in wheat. Plant Growth Regul. 2001;35: 239–245. 10.1023/A:1014411316780

[21] Williams RH, Cartwright PM. The effect of applications of a synthetic cytokinin on shoot dominance and grain yield in spring barley. Ann Bot. 1980;46: 445–452.

[22] Cheng X, Ni H. Weed control efficacy and winter wheat safety of a novel herbicide HW02. Crop Protect. 2013;43: 246–250. 10.1016/j.cropro.2012.10.016

[23] Leaden MI, Lozano CM, Monterubbianesi MG, Abello EV. Spring wheat tolerance to DE-750 applications at different growth stages. Weed Technol. 2007;21: 406–410. 10.1614/WT-06-102R1.1

[24] Zhang J, Liu W, Yang X, Gao A, Li X, Wu X, et al. Isolation and characterization of two putative cytokinin oxidase genes related to grain number per spike phenotype in wheat. Mol Biol Rep. 2011;38: 2337–2347. 10.1007/s11033-010-0367-9 21104150

[25] Serrago RA, Miralles DJ, Slafer GA. Floret fertility in wheat as affected by photoperiod during stem elongation and removal of spikelets at booting. Eur J Agron. 2008;28: 301–308. 10.1016/j.eja.2007.08.004

[26] Shi RQ, Xie HL, Li PS, Zang HQ. Effects of spraying exterior substance after florescence on wheat hormone contents and source-sink modulation. Journal of Henan Agricultural University. 2006;40: 122–126.

[27] González FG, Slafer GA, Miralles DJ. Floret development and spike growth as affected by photoperiod during stem elongation in wheat. Field Crops Res. 2003;81: 29–38. 10.1016/S0378-4290(02)00196-X

[28] Waddington SR, Cartwright PM, Wall PC. A quantitative scale of spike initial and pistil development in barley and wheat. Ann Bot. 1983;51: 119–130.

[29] Li C, Cao W, Dai T. Dynamic characteristics of floret primordium development in wheat. Field Crops Res. 2001;71: 71–76. 10.1016/S0378-4290(01)00144-7

[30] Li CD, Cao WX, Dai TB, Yan MC, Wang ZL. Study on dynamic models and characteristics of floret primordium differentiation and degeneration in wheat. Scientia Agricultura Sinica. 1999;32: 98–100.

[31] Prystupa P, Savin R, Slafer GA. Grain number and its relationship with dry matter, N and P in the spikes at heading in response to N×P fertilization in barley. Field Crops Res. 2004;90: 245–254. 10.1016/j.fcr.2004.03.001

[32] Slafer GA, Abeledo LG, Miralles DJ, Gonzalez FG, Whitechurch EM. Photoperiod sensitivity during stem elongation as an avenue to raise potential yield in wheat. Euphytica. 2001;119: 191–197. 10.1023/A:1017535632171

[33] Wang ZL, Cao WX, Dai TB, Zhou Q. Characteristics of floret development and grain set in three wheat genotypes of different spike sizes. Journal of Nanjing Agricultural University. 2000;23: 9–12.

[34] Wang Z, Cao W, Dai T, Zhou Q. Effects of exogenous hormones on floret development and grain set in wheat. Plant Growth Regul. 2001;35: 225–231. 10.1023/A:1014442006862

[35] Peltonen-Sainio P. Nitrogen fertilizer and foliar application of cytokinin affect spikelet and floret set and survival in oat. Field Crops Res. 1997;49: 169–176. 10.1016/S0378-4290(96)01010-6

[36] Miralles DJ, Katz SD, Colloca A, Slafer GA. Floret development in near isogenic wheat lines differing in plant height. Field Crops Res. 1998;59: 21–30. 10.1016/S0378-4290(98)00103-8

[37] Slafer GA, Araus JL, Royo C, Moral LFG. Promising eco-physiological traits for genetic improvement of cereal yields in Mediterranean environments. Ann Appl Biol. 2005;146: 61–70. 10.1111/j.1744-7348.2005.04048.x

[38] Arisnabarreta S, Miralles DJ. Floret development and grain setting in near isogenic two- and six-rowed barley lines (Hordeum vulgare L.). Field Crops Res. 2006;96: 466–476. 10.1016/j.fcr.2005.09.004

[39] Norimoto M. Plant growth hormone cytokinins control the crop seed yield. Am J Plant Sci. 2014;5: 2178–2187.

[40] Mok DW, Mok MC. Cytokinin metabolism and action. Annu Rev Plant Physiol Plant Mol Biol. 2001;52: 89–118. 10.1146/annurev.arplant.52.1.89 11337393

[41] Bartrina I, Otto E, Strnad M, Werner T, Schmülling T. Cytokinin regulates the activity of reproductive meristems, flower organ size, ovule formation, and thus seed yield in Arabidopsis thaliana. Plant Cell. 2011;23: 69–80. 10.1105/tpc.110.079079 21224426 PMC3051259

[42] Ashikari M, Sakakibara H, Lin S, Yamamoto T, Takashi T, Nishimura A, et al. Cytokinin oxidase regulates rice grain production. Science. 2005;309: 741–745. 10.1126/science.1113373 15976269

[43] Rijavec T, Kovac M, Kladnik A, Chourey PS, Dermastia M. A comparative study on the role of cytokinins in caryopsis development in the maize miniature1 seed mutant and its wild type. J Integr Plant Biol. 2009;51: 840–849. 10.1111/j.1744-7909.2009.00863.x 19723243

[44] Zalewski W, Galuszka P, Gasparis S, Orczyk W, Nadolska-Orczyk A. Silencing of the HvCKX1 gene decreases the cytokinin oxidase/dehydrogenase level in barley and leads to higher plant productivity. J Exp Bot. 2010;61: 1839–1851. 10.1093/jxb/erq052 20335409

